# Enhancement of lemongrass essential oil physicochemical properties and antibacterial activity by encapsulation in zein-caseinate nanocomposite

**DOI:** 10.1038/s41598-024-67273-6

**Published:** 2024-07-27

**Authors:** Sara A. Alsakhawy, Hoda H. Baghdadi, Moustafa A. El-Shenawy, Lobna S. El-Hosseiny

**Affiliations:** 1 Department of Environmental Studies, Institute of Graduate Studies and Research, Alexandria University, Alexandria, 21526 Egypt; 2 Department of Food Microbiology, National Research Center, Dokki, Cairo, 12311 Egypt

**Keywords:** Zein nanoparticles, Sodium caseinate, Lemongrass essential oil, Polymeric drug delivery system, Antimicrobial activity, Citral, Microbiology, Nanoscience and technology

## Abstract

Essential oils (EOs) represent a pivotal source for developing potent antimicrobial drugs. However, EOs have seldom found their way to the pharmaceutical market due to their instability and low bioavailability. Nanoencapsulation is an auspicious strategy that may circumvent these limitations. In the current study, lemongrass essential oil (LGO) was encapsulated in zein-sodium caseinate nanoparticles (Z-NaCAS NPs). The fabricated nanocomposite was characterized using dynamic light scattering, Fourier-transform infrared spectroscopy, differential scanning calorimetry, and transmission electron microscopy. The antimicrobial activity of LGO loaded NPs was assessed in comparison to free LGO against *Staphylococcus epidermidis, Enterococcus faecalis, Escherichia coli,* and *Klebsiella pneumoniae*. Furthermore, their antibacterial mechanism was examined by alkaline phosphatase, lactate dehydrogenase, bacterial DNA and protein assays, and scanning electron microscopy. Results confirmed the successful encapsulation of LGO with particle size of 243 nm, zeta potential of – 32 mV, and encapsulation efficiency of 84.7%. Additionally, the encapsulated LGO showed an enhanced thermal stability and a sustained release pattern. Furthermore, LGO loaded NPs exhibited substantial antibacterial activity, with a significant 2 to 4 fold increase in cell wall permeability and intracellular enzymes leakage versus free LGO. Accordingly, nanoencapsulation in Z-NaCAS NPs improved LGO physicochemical and antimicrobial properties, expanding their scope of pharmaceutical applications.

## Introduction

Infectious diseases are rated as one of the most menacing healthcare issues of the 21st century, particularly with the indiscriminate use of conventional antibiotics, which has resulted in the development of drug-resistant bacteria, prolonged patient illness, and increased mortality rates^1^. Several therapeutic approaches have recently been developed to combat bacterial infections, including metal nanoparticles (NPs), polymeric NPs, and natural bioactives^2,3^. Nevertheless, metal nanoparticles exhibit high cytotoxicity and a tendency to bioaccumulate in sensitive organs, resulting in limited clinical applications^4^. Alternatively, polymer based nanoparticles and natural bioactives are potential candidates for fabricating biocompatible, efficient antimicrobial based nanoplatforms^5^. Natural bioactives, in particular essential oils derived from aromatic plants, are constituted by an array of complex volatile organic components, comprising terpenes, terpenoids, and phenols, which confer them immense biological activities, including antibacterial, antifungal, antiviral, anticancer, and antioxidant properties^6^. Furthermore, essential oils phytochemical diversity privileges their potential in minimizing bacterial resistance development as compared to synthetic antimicrobials^7^. However, essential oil-based antimicrobials have seldom found their way to the pharmaceutical market since the advent of antibiotics in 1950s, due to their poor aqueous solubility, low bioavailability, high volatility, sensitivity to various environmental factors, as well as high risk of instability and bioactive deterioration^8^.

Within the framework of nanotechnological developments, polymer based nanoencapsulation of essential oils appeals as an auspicious approach to provide better protection of their volatile constituents, improve their bioavailability, bioefficacy, and sustain the release of their bioactive constituents^9,10^. Various methods of nanoencapsulation have been explored in this regard; nonetheless, nanoencapsulation utilizing natural polymers has gained special interest over synthetic ones due to their biocompatibility, availability, cost-effectiveness, and biodegradability^11^. Protein based NPs, in particular, demonstrated several advantages for encapsulating natural bioactives owing to their high encapsulating capacity, stability, ease of preparation, and credibility of production at a large scale^12^. In addition, protein biopolymers have multiple functional groups, which allow them to create diverse interactions with molecules of different polarities based on electrostatic interactions and hydrophobic bonding^13^. Furthermore, as compared to polysaccharide based NPs, protein biopolymers demonstrate the capability of developing small-sized NPs with improved cell penetration potential^14^.

Zein (Z), a plant protein extracted from maize, has been considered a generally regarded as safe (GRAS) polymer by the food and drug administration (FDA). Zein presents a propitious candidate for natural bioactive encapsulation owing to its hydrophobic nature, low cost, high safety profile, and controllable drug release properties^15^. Several methods have been adopted to synthesize Z NPs, including electrospraying, anti-solvent precipitation, and solvent emulsification-evaporation. The anti-solvent precipitation method is the most widely used approach for Z NPs synthesis due to its simplicity, reproducibility, and eco-friendliness. In addition, the anti-solvent precipitation method has the potential to fabricate Z NPs with a homogeneous particle size and a high encapsulation rate^16^. However, there are considerable challenges regarding the fabrication of Z NPs via anti-solvent precipitation method, including poor colloidal stability, tendency for aggregation and precipitation at neutral pH, and subsequent functionality loss. Accordingly, better control of Z NPs dispersion stability and aqueous solubility should be optimized to broaden their scope of application. Introducing an amphiphilic biopolymer during the anti-solvent preparation procedure of Z NPs is an impending way to reduce their surface hydrophobicity, improve their stability, and endow new functional properties^17^. Sodium caseinate (NaCAS), an amphiphilic GRAS protein biopolymer, possesses exceptional attributes, including surface active and stabilizing properties, emulsification and self-assembly characteristics, as well as water binding capacity, which puts it forth as an adequate candidate to overcome Z NPs undesirable properties^18^. The NaCAS biopolymer is proposed to have a dual role, where its hydrophobic part is adsorbed on Z NPs via hydrophobic interaction. Meanwhile, its hydrophilic hairy layer improves Z NPs aqueous solubility and physical stability via electrostatic repulsion^19^.

Among the vastness of plant extracts, lemongrass essential oil (LGO), extracted from *Cymbopogon* species, has captivated interest due to its FDA-approved safety and enrichment with valuable phytoconstituents of propitious pharmacological properties, including antimicrobial potential^20^. However, LGO hydrophobicity, high volatility, and short half-life impede its clinical applications. Nanoencapsulation in biopolymers may circumvent these limitations. To the best of our knowledge, nanoencapsulation of LGO in Z based nanoparticulate systems hasn’t been investigated yet. Moreover, the antibacterial mechanism of LGO loaded nanoparticulate systems has not been documented to date. Accordingly, the present study aimed to develop a novel nanodelivery system for LGO based on Z and NaCAS biopolymers and assess its antimicrobial potential in comparison to unencapsulated LGO. The fabricated LGO loaded Z-NaCAS NPs were characterized physicochemically, morphologically, and thermally. The in vitro release properties and release kinetics of LGO loaded NPs were examined. Furthermore, the antibacterial potential of LGO loaded Z-NaCAS NPs, as well as their potential mechanism of action, was investigated against two Gram-positive and two Gram-negative bacteria.

## Results and discussion

### Phytochemical composition of LGO

**Figure 1:**
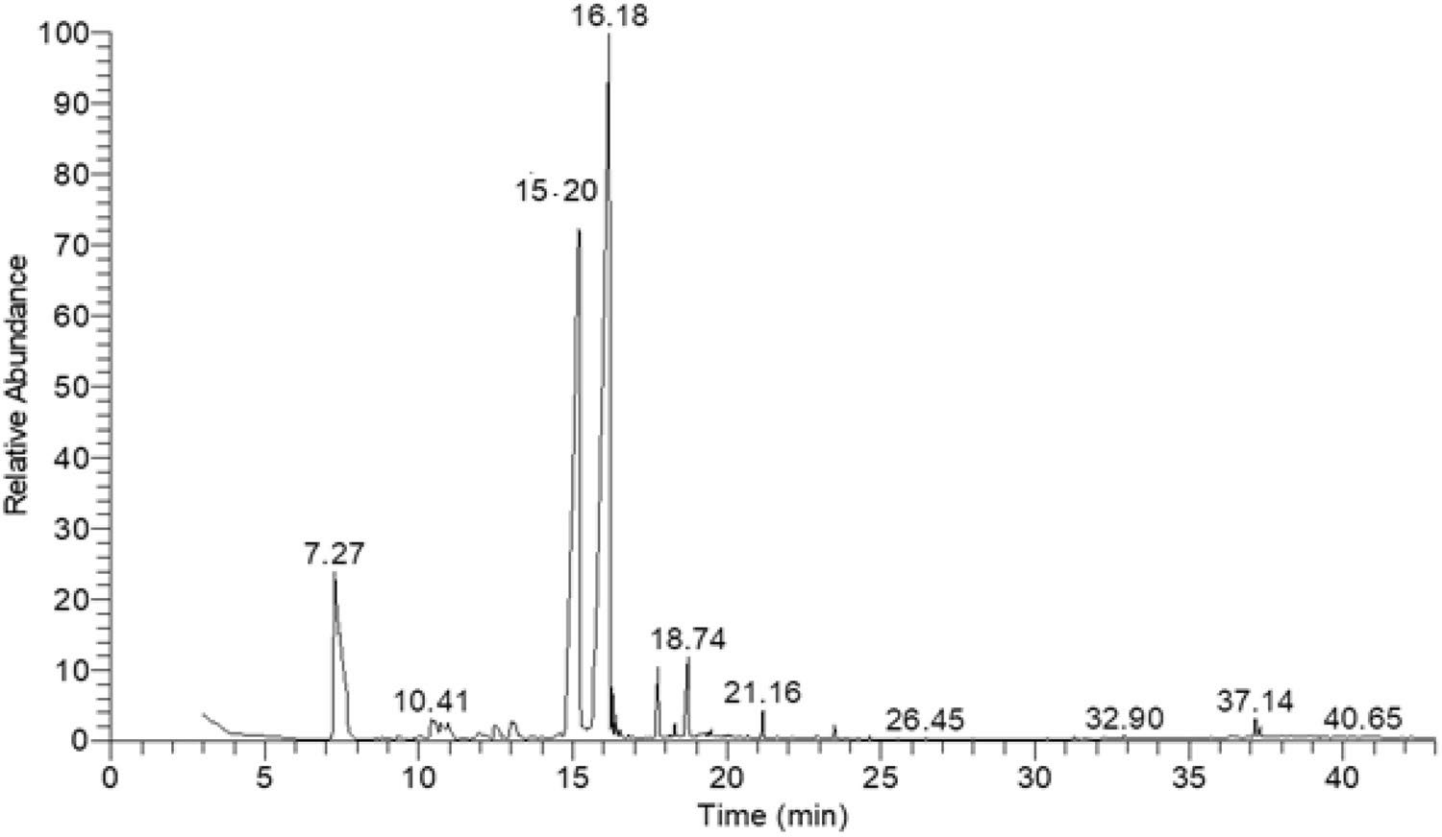
GC–MS chromatogram of lemongrass essential oil.

The hydrodistilled LGO was constituted mainly by monoterpenes and sesquiterpenes, accounting for 90% of the essential oil composition. As depicted in Fig. 1 and Table 1, the acyclic monoterpene aldehyde citral, comprising 70.22% of the oil composition, was the major component. The second most abundant constituent was myrecene (13.64%), followed by caryophyllene and its oxide, comprising 0.36% and 1.18%, respectively. Meanwhile, trace amounts of the alcoholic terpenes geraniol, myrecenol, linalool, and verbenol were also present, constituting 0.35%, 0.91%, 1.21%, and 1.74%, correspondingly. The present results are in accordance with previous studies reporting citral as the main constituent of LGO with a relative abundance of 65–85%^21–23^. Citral concentration in LGO is an important quality indicator for the extracted essential oil, contributing to its bioactivity^24^. It is reported that citral is a combination of the two aldehydic isomers geranial (α-citral) and neral (β-citral), accounting for 40–62% and 25–38%, respectively, which is in accordance with the present results since the trans isomer geranial was dominant over the cis isomer neral^20^.

**Table 1:** Phytochemical composition of lemongrass essential oil.

Component	Retention time (min)	Percentage (%)
Myrcene	7.27	13.64%
Myrecenol	10.41	0.91%
Linalool	10.68	1.21%
Verbenol	12.44	1.74%
Neral (β-citral)	15.22	28.03%
Geranial (α-citral)	16.18	42.19%
Caryophyllene oxide	18.74	1.18%
Caryophyllene	21.16	0.36%
Geraniol	26.45	0.35%

### Characterization of LGO loaded Z-NaCAS NPs

#### Fourier-transform infrared spectroscopic analysis

**Figure 2:**
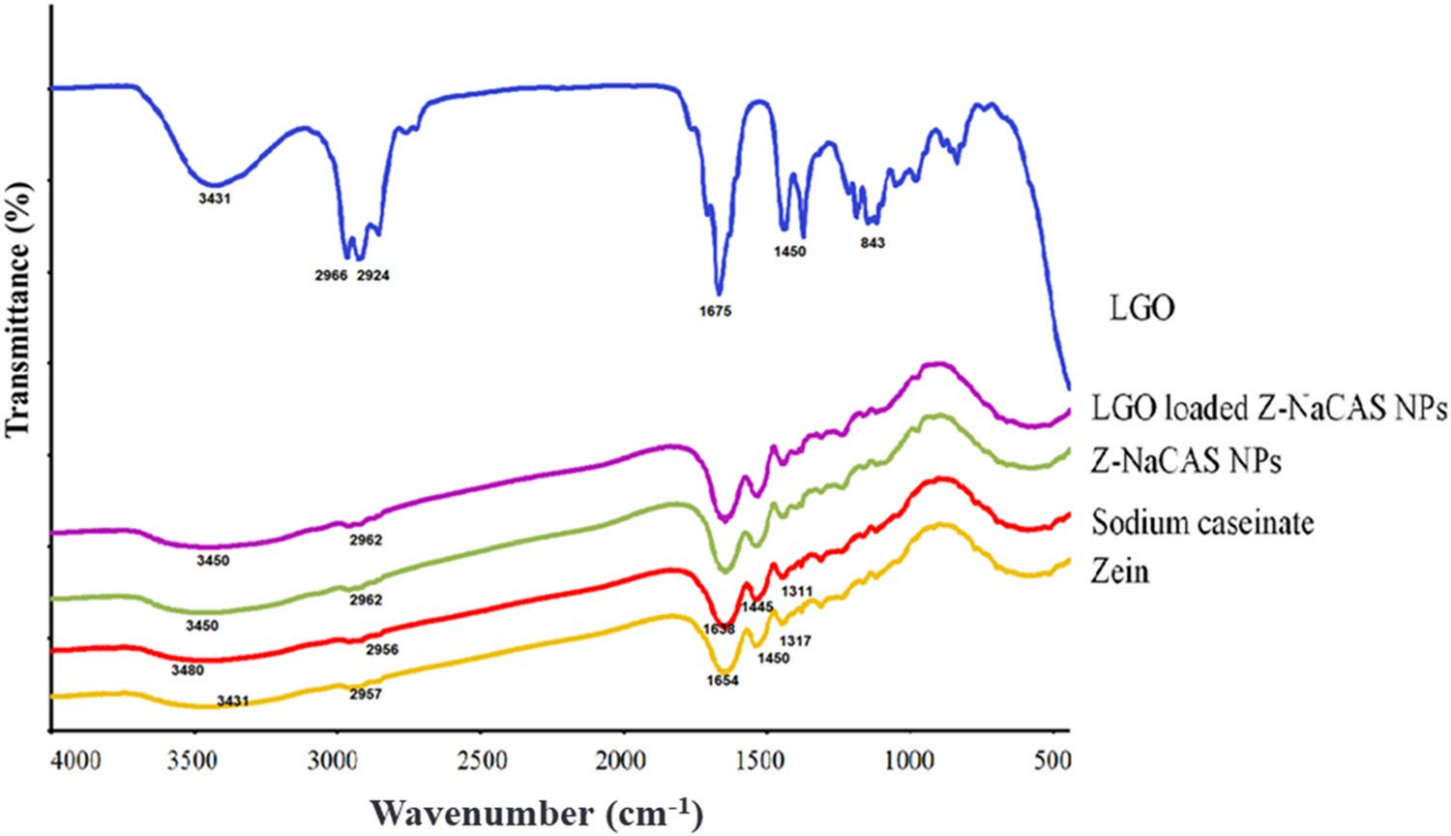
FTIR of Z, NaCAS, LGO, blank Z-NaCAS NPs and LGO loaded Z-NaCAS NPs.

The FTIR analysis was carried out to investigate the chemical structure and the possible intermolecular interactions between Z, NaCAS, and LGO (Fig. 2). The spectrum of free LGO demonstrated a broad peak at 3431 cm^−1^, corresponding to the hydroxyl group, as well as another branched peak at 2966–2924 cm^−1^ due to the C-H stretching of aliphatic alkyl groups^25^. In addition, a sharp characteristic absorption peak was observed at 1675 cm^−1^ corresponding to the C = O stretching vibrations of aldehydes, mainly citral. In addition, absorption peaks at 1450 and 843 cm^−1^ were assigned to the –CH_3_ and C–H vibrations of benzene rings, respectively^26,27^.

In the spectra of Z and NaCAS, typical peaks at 3431–3480 cm^−1^ and 2957—2965 cm^−1^ were detected, respectively, corresponding to the hydrophilic O–H stretching and the hydrophobic C–H group^28^. In addition, the distinctive peaks for amide I, II, and III bonds were observed in the Z spectrum at 1654, 1450 and 1317 cm^−1^, respectively and at 1638, 1445, and 1311 cm^−1^ in the NaCAS spectrum^29^. Regarding the FTIR spectrum of blank Z-NaCAS NPs, it was found that upon interaction of Z with NaCAS, the peak of OH shifted to 3450 cm ^−1^, inferring the formation of hydrogen bonds between the amide groups of Z and the hydroxyl groups of NaCAS. However, in the LGO loaded Z-NaCAS NPs spectrum, it was found that nanoencapsulation of LGO did not change the peaks of amide I and II in Z and NaCAS polymers; moreover, most of the LGO peaks disappeared, indicating that no chemical reaction occurred and non-covalent interactions were mainly involved between LGO and Z during the nanoencapsulation process^30^. Similar results were reported in previous studies nanoencapsulating curcumin, naringin, tocopherrol, resveratrol, and fucoxanthin in Z based NPs^31–34^.

#### Differential scanning calorimetry analysis

**Figure 3:**
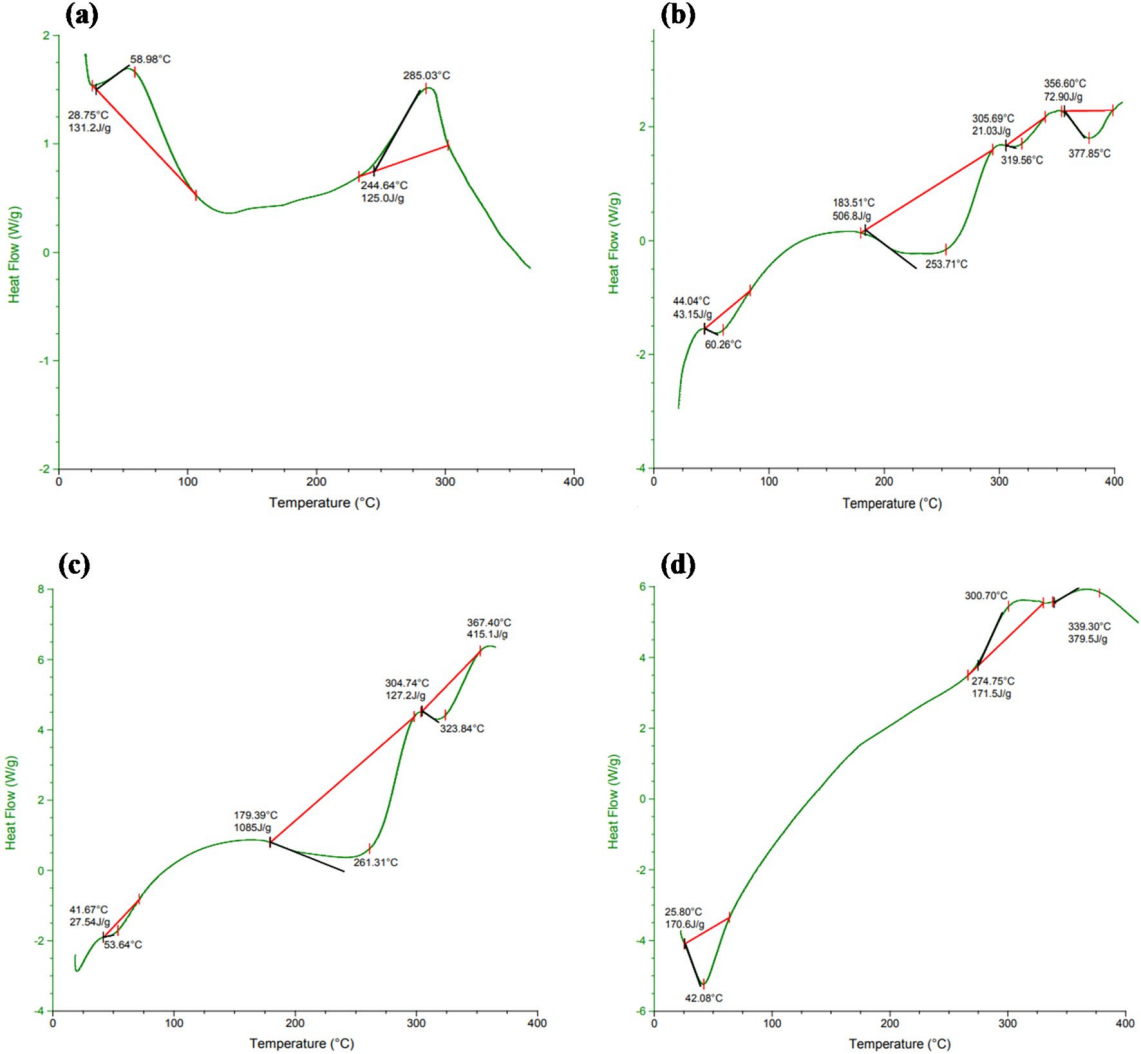
DSC of Z (**a**), NaCAS (**b**), blank Z-NaCAS NPs (**c**) and LGO loaded Z-NaCAS NPs (**d**).

The thermal stability of Z, NaCAS, blank Z-NaCAS NPs, and LGO loaded Z-NaCAS NPs was investigated using DSC. As shown in Fig. 3, the DSC thermograms of Z and NaCAS showed a small peak near 60 °C, which corresponds to their first weight loss due to the evaporation of water from their surfaces. Then, a sharp peak appeared at 285.03 and 253.71 °C, which is ascribed to the second weight loss due to the thermal decomposition of Z and NaCAS, respectively^35,36^. In blank Z-NaCAS NPs, only one degradation peak was obtained at 261.31°C, inferring the compatibility between Z and NaCAS and possible strong hydrogen and hydrophobic interactions between them leading to more compact NPs^37^. Compared to blank NPs, LGO loaded Z-NaCAS NPs demonstrated a new degradation peak at 300.70 °C, reflecting a significant enhancement in the thermal stability of encapsulated LGO as well as the successful encapsulation of LGO within the hydrophobic core of Z-NaCAS NPs in an amorphous state. The present results are consistent with a study that reported an improvement in the thermal stability of peppermint and green tea essential oils upon encapsulation in chitosan NPs^38^.

#### Dynamic light scattering analysis

**Figure 4:**
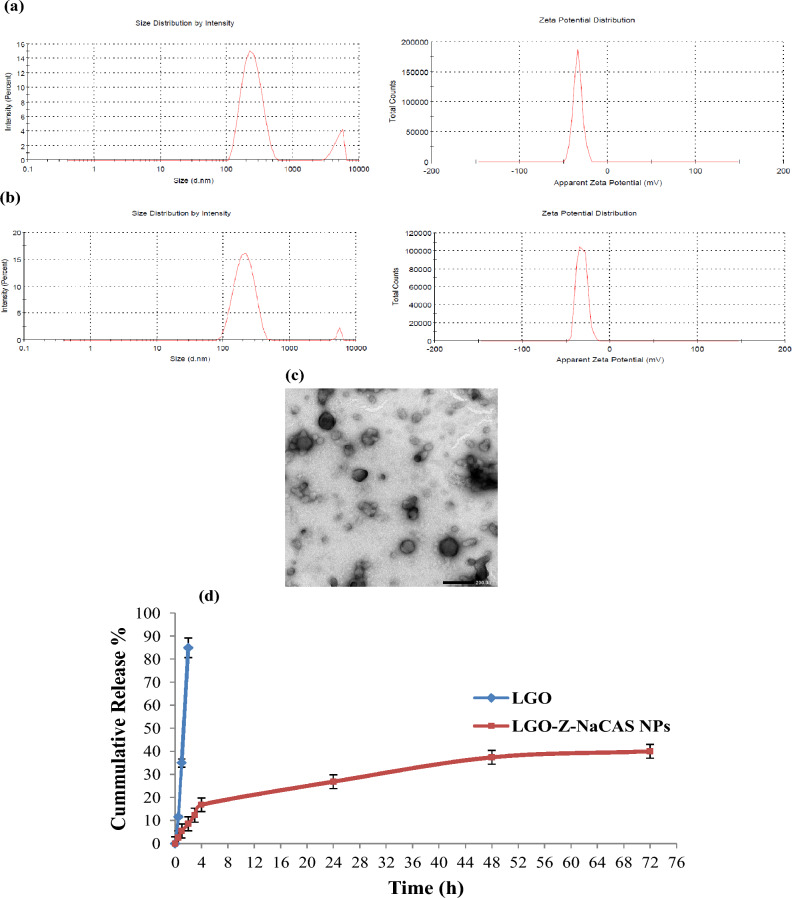
Average particle size and zeta potential distributions of blank Z-NaCAS NPs (**a**) and LGO loaded Z-NaCAS NPs (**b**), TEM image of LGO loaded Z-NaCAS NPs (**c**), in vitro release profiles of free LGO and LGO loaded Z-NaCAS NPs (**d**).

In the current investigation, blank Z-NaCAS NPs exhibited an average particle size and zeta potential (ZP) of 232 ± 70.52 nm and − 34.1 mV, compared to 243 ± 68.29 nm and −32.1 mV for LGO loaded Z-NaCAS NPs (Fig. 4a and b). As shown, following LGO encapsulation, there were no significant changes in particle size or ZP. These insignificant differences may be attributed to the inclusion of LGO in the hydrophobic core of Z, thereby limiting the impact of LGO on size and surface characteristics. In addition, the higher negative ZP values of the fabricated NPs infer greater electrostatic repulsion at their surfaces, resulting in their colloidal stability. As is acknowledged, ZP values greater than ± 30 mV suggest high stability, while values near ± 20 mV indicate short-term stability, and those less than ± 5 mV indicate low stability and rapid aggregation of NPs^38^. Meanwhile, the negative ZP values of the fabricated NPs may be attributed to the phosphate ester and carboxylate groups of NaCAS, which act as an electrostatic stabilizer of Z NPs, preventing their spontaneous aggregation into large particles^39^. Furthermore, both blank and LGO loaded Z-NaCAS NPs showed PDI values below 0.4, inferring their homogenous particle size distributions, as it is reported that the PDI value is an estimate of NPs size distribution uniformity, with low PDI values indicating monodispersity and greater stability^40^.

#### Encapsulation efficiency and loading capacity

Encapsulation efficiency and loading capacity are essential properties of nanoencapsulation that reflect the level of drug loading into NPs. In the present study, the fabricated LGO loaded Z-NaCAS NPs exhibited an EE% of 84.7 ± 5% and LC% of 9.2 (w/w). This high EE% and LC% results may be attributed to the hydrophobic nature of LGO and the synergy of the Z hydrophobic core, as well as the coating layer of NaCAS on the Z NPs surface, which limits the outward diffusion of LGO^29^. Similar findings were reported in previous studies, which showed an improvement in the EE% of Z NPs upon coating with an additional layer of polymer such as pectin, chitosan, and gum arabic^41–43^.

#### Transmission electron microscopy analysis

The morphological features of LGO loaded Z-NaCAS NPs were characterized by TEM. As shown in Fig. 4c, the fabricated NPs were spherical in shape with a uniform structure. Similar morphological characteristics were also reported by other researchers for rutin, carvacrol, and naringin encapsulated in Z-NaCAS NPs^29,34,44^. The mean particle size of LGO loaded Z-NaCAS NPs was around 108 ± 16 nm, which is comparatively smaller than the average particle size obtained by DLS measurement. This difference may be ascribed to the physical state of the tested NPs sample, where the particle size measurement in TEM is performed in the dry matter state, while in DLS analysis, its measurement is carried out in liquid suspension and is mainly linked to their hydrodynamic diameter in the solvated state^38^.

#### In vitro release kinetics of LGO loaded Z-NaCAS NPs

The in vitro release profiles of free LGO and LGO loaded Z-NaCAS NPs were investigated using dialysis bag method at pH 7.4. As shown in Fig. 4d, unencapsulated LGO exhibited a rapid diffusion rate, and almost 85% of LGO was released after 2 h from contact with the release medium. On the contrary, the LGO encapsulated in Z-NaCAS NPs demonstrated a much slower release profile with a biphasic pattern. At first, there was an initial burst release for 5 h, and up to 20% of LGO was released. This fast, early release may be attributed to the rapid diffusion of LGO localized at the surface layer of Z NPs^45^. In the second phase, a sustained release of LGO was observed, with a total accumulative release of 27, 37, and 40% after 24, 48, and 72 h, respectively. This slow, gradual release may be ascribed to the degradation of Z-NaCAS NPs, resulting in the diffusion of entrapped LGO into the release medium^46^. The present results are coherent with previous studies reporting the biphasic release pattern of β-carotene, 7,8-dihydroxyflavone, and naringin from Z coated NPs^34,47,48^.

To analyze the release kinetics of LGO from Z-NaCAS NPs, the drug release profile was fitted into different release kinetic models (zero-order, first-order, Korsmeyer Peppas, Higuchi, and Hixson-Crowell models) and evaluated using the correlation coefficient R2. As shown in Supplementary Table S1 and Supplementary Figure S1, the release profile of LGO from Z-NaCAS NPs followed Higuchi's model as it showed the highest correlation coefficient (R2 = 0.98). Accordingly, based on the Higuchi model, the current findings indicate that the release kinetics of LGO from Z-NaCAS NPs follow the diffusion mechanism and the slow degradation of polymeric NPs^49,50^. The drug release diffusion type was anticipated from the obtained n value (release exponent) of Korsmeyer–Peppas model. The current results show that the n value ≤ 0.45, indicating fickian diffusion^51^. Accordingly, it can be proposed that the fabricated Z-NaCAS NPs are a promising nanodelivery system since they sustain the release of encapsulated LGO.

#### Antibacterial activity

The antibacterial activity of unencapsulated LGO, blank Z-NaCAS NPs, and LGO loaded Z-NaCAS NPs was evaluated using broth microdilution assay against *Staphylococcus epidermidis, Enterococcus faecalis, Escherichia coli*, and *Klebsiella pneumoniae*. The current results revealed that blank Z-NaCAS NPs did not exhibit any inhibitory activity against all the tested organisms. In contrast, both free and nanoencapsulated LGO exhibited substantial antibacterial activity against all the tested bacterial strains, with MIC values of 0.8 mg/mL. Similar findings were reported by previous studies, where no difference was indicated in the MIC values of free and nanoencapsulated essential oils^39,52,53^.

#### Antibacterial mechanism of action

##### Bacterial cells morphology

**Figure 5:**
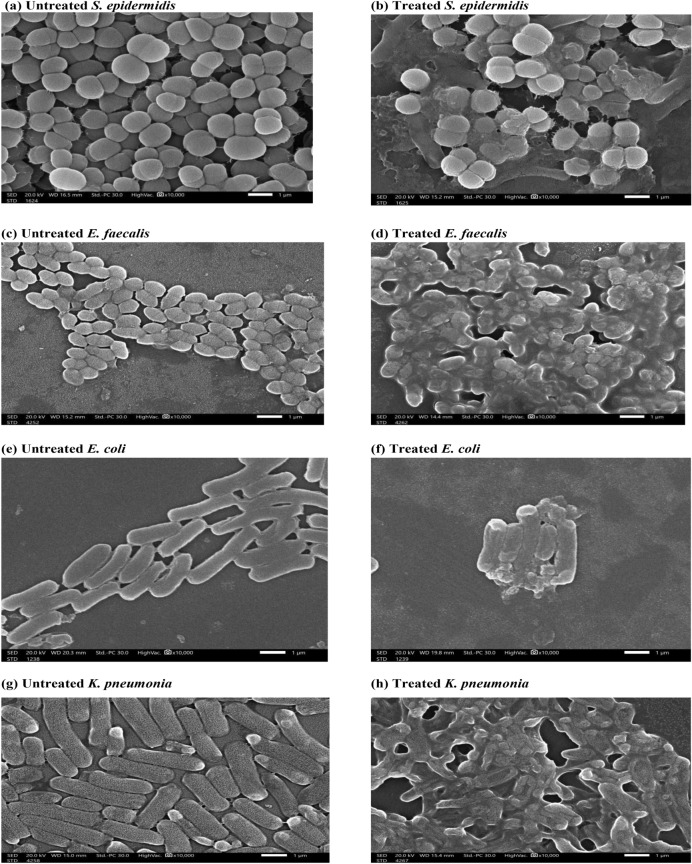
Scanning electron micrographs of untreated and 1 MIC LGO loaded Z-NaCAS NPs treated bacterial cells.

Apparent morphological changes were observed in bacterial cells treated with 1 MIC of LGO loaded Z-NaCAS NPs, as revealed by SEM examination. Control untreated bacterial cells exhibited the normal morphological characteristics of the corresponding tested bacterial strain, and the cell surface was smooth and intact. However, treated cells showed significant changes where the typical spherical, rod, or coccobacillary shape was deformed and the cell surface shifted to a pitted, shriveled, and rugged topography (Fig. 5). The present results are in accordance with a study that reported a morphological alteration of *S. aureus* bacteria upon treatment with citral, the major LGO phytoconstituent^54^. The observed morphological changes of treated bacteria are probably related to changes in the cell envelope, which was further investigated via assays of proteins and nucleic acid leakage, as well as LDH and AKP activity assays.

##### Bacterial proteins and nucleic acids leakage

**Figure 6:**
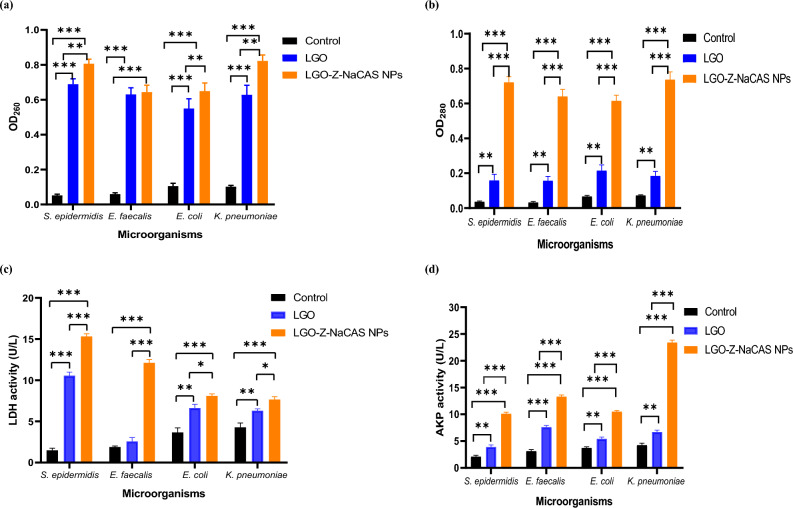
Nucleic acids (**a**), proteins (**b**), lactate dehydrogenase (**c**) and alkaline phosphatase (**d**) release from control, LGO and LGO loaded Z-NaCAS NPs treated bacterial cells. Data are expressed as means ± SD, graph asterisks denote statistical significance at *P < 0.05, **P < 0.01 and ***P < 0.001.

The potential mode of action of free unencapsulated LGO and LGO loaded Z-NaCAS NPs on bacterial cells was assessed, and it was found that the absorbance of cellular components at 260 and 280 nm was significantly higher for both LGO and LGO loaded Z-NaCAS NPs treated bacterial cells as compared to control untreated cells (Fig. 6a and b). The OD 260 and 280 nm represent the maximum absorption peaks of nucleic acid and protein and indicate the effect of the tested agent on bacterial cell membrane integrity, where high absorption values infer nucleic acids and proteins leakage from bacterial cells^55^. The present results showed that after treatment of bacterial cells with 1 MIC of unencapsulated LGO, the OD260 nm increased by 10.9 and 10.5 versus 6.2 and 6.6 folds for *Staphylococcus epidermidis, Enterococcus faecalis, Escherichia coli*, and *Klebsiella pneumoniae*, respectively. Meanwhile, 1 MIC nanoencapsulated LGO treated cells demonstrated a significant increase in OD260 nm by 12.9, 11.6, 8.1, and 9.2 folds as compared to free LGO treated cells, correspondingly.

Regarding the maximum absorption peak for proteins, there was also a significant increase in OD 280 nm in LGO loaded Z-NaCAS NPs treated bacterial cells by 17.1, 21, 6.9, and 10.8 folds as compared to free LGO treated cells that showed an increase by 5.8, 6.7, 3.1, and 2.7 folds for *S. epidermidis, E. faecalis, E. coli*, and* K. pneumoniae*, respectively. In context of the present results, the influence of either free or LGO loaded Z-NaCAS NPs was markedly higher in tested Gram-positive bacteria as compared to Gram-negatives. In general, the antimicrobial actions of essential oils vary according to the essential oil as well as the bacterial strain tested. Essential oils react differently on Gram-positive and Gram-negative bacteria owing to their dissimilar cell wall structures^56^. It has been depicted that the entry of essential oils into bacterial cells is eased in Gram-positive bacteria owing to the absence of a rigid exoskeleton peptidoglycan layer, which may explain the markedly higher influence of LGO and LGO loaded Z-NaCAS NPs on OD 260 and 280 cellular components in Gram-positive bacteria. Several studies reported similar results in which Gram-positive bacteria exhibited higher sensitivity to the tested essential oils owing to their hydrophobic nature, which restricted their interaction with the hydrophilic lipopolysaccharides distributed on the outer membrane surface of Gram-negative bacteria^57–61^.

##### Lactate dehydrogenase activity

Besides assessing the leakage of proteins and nucleic acids as indicators of the potential destructive effect of LGO on bacterial cell membrane, LDH was also investigated as a marker for cell membrane disruption. Treatment of *S. epidermidis, E. faecalis, E. coli,* and* K. pneumoniae* cells with 1 MIC of LGO loaded Z-NaCAS NPs resulted in a significant induction of LDH levels by 11.5%, 6.1%, 1.7%, and 1%, respectively, which is approximately double that induced by free LGO (Fig. 6c). Lactate dehydrogenase is a glycolytic enzyme located in the cytosol of most organisms; hence, its leakage is a marker for the loss of membrane integrity^62^. The increase in the activity of LDH may also be related to the oxidative stress condition in bacterial cells, which is associated with disruption of cell integrity during lipid peroxidation, hence leading to an increase in the level of lactic acid and lactic acid salts^63^.

##### Alkaline phosphatase activity

**Figure 7:**
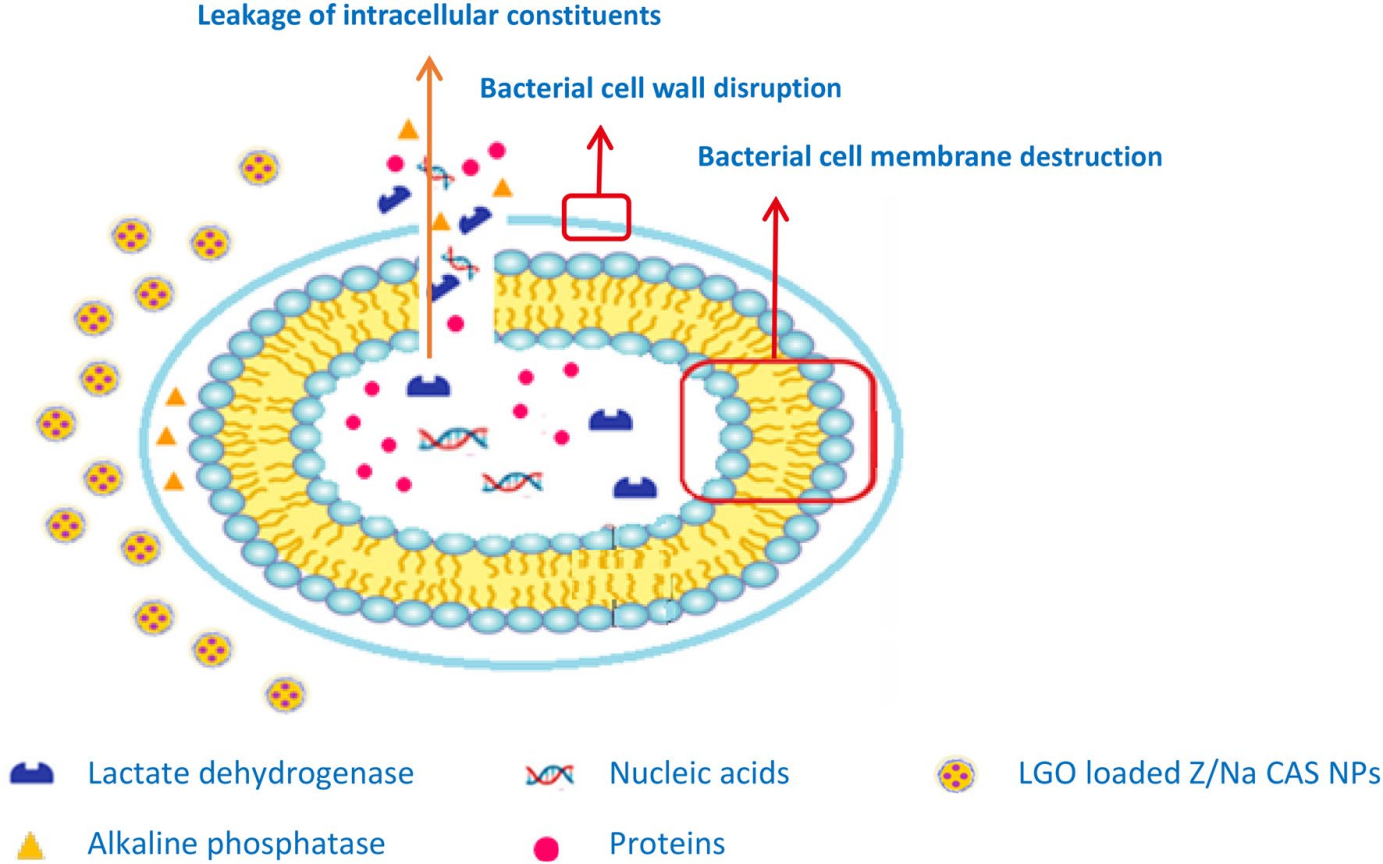
Schematic illustration of the antibacterial mechanisms of LGO loaded Z-NaCAS NPs.

In the present study, upon examining the influence of free LGO and LGO loaded Z-NaCAS NPs on AKP as an indicator of cell wall integrity, results demonstrated that treatment of bacterial cells with 1 MIC of free LGO or LGO loaded Z-NaCAS nanocomposite caused a significant increase in AKP concentration as compared to untreated cells. In addition, bacterial cells treated with LGO loaded Z-NaCAS NPs exhibited a significant three-fold increase in AKP as compared to free LGO treated cells (Fig. 6d). Accordingly, it can be proposed that an increase in the extracellular concentration of AKP signifies cell wall disruption owing to the location of this enzyme in the periplasmic space and its eventual leakage following cell wall damage^64^. In addition, AKP leakage has also been linked to the stress imposed on bacterial cells, since it is reported that the production of AKP increases during phosphate starvation associated with stress to generate free phosphate groups for uptake and use^65^. Eventually, the increase in AKP in treated cells may, on the one hand, be related to cell wall damage induced by LGO or to the increased expression of AKP in treated cells to circumvent the stress induced by exposure to the essential oil. To the best of our knowledge, the antibacterial mechanism of nanoencapsulated LGO in polymeric NPs has not been investigated yet; hence, it can be proposed that the fabricated LGO loaded Z-NaCAS NPs exert their antibacterial effect via bacterial cell wall destruction, cell membrane disruption, and vital enzyme alteration, eventually inducing bacterial cell death (Fig. 7). The current antibacterial potentiality of free and nanoencapsulated LGO may be ascribed to citral, the major phytoconstituent of LGO, which has been reported to disrupt membrane fluidity in *S. aureus*^54^.

In context of the present results, LGO loaded Z-NaCAS NPs displayed a significantly higher effect on bacterial cell membrane integrity, cell wall permeability, and enzyme activity as compared to free LGO. Similar findings were reported in other studies where thymol and quercetin encapsulated in Z NPs displayed improved antibacterial properties as compared to the unencapsulated phytoconstituents^60,66^. Hence, nanoencapsulation in Z based NPs proposedly enhanced the bioactivity by facilitating transport across the bacterial cell membrane and prolonging contact with the target site in bacterial cells. On the contrary, Soltanzadeh et al*.*^26^ reported that encapsulation of LGO in the polysaccharide based polymer chitosan resulted in significantly lower antimicrobial activity as compared to free LGO. Accordingly, it can be conceived that the polymer type used for encapsulation of essential oils plays a significant role in maintaining their bioactivity. In addition, the biocompatibility, biodegradability, and non-toxicity of the nanodelivery system are crucial factors too. The cytocompatibility of Z based nanoformulations has been reported in several studies, as well as that of LGO in collagen-chitosan membranes^34,67,68^. It’s worth mentioning that in the present study, the LGO concentration used for antimicrobial assessment (0.8 mg/mL) is considerably lower than the reported cytotoxic concentration of LGO ^68^.

## Conclusion

The present study developed a novel biocompatible Z-NaCAS nanodelivery system for LGO utilizing the simple and cost effective anti-solvent precipitation method. The fabricated LGO loaded NPs exhibited spherical structure with average particle size of 243 nm, ZP of – 32 mV, EE% of 84.7%, and LC% of 9.2 (w/w). The FTIR analysis revealed that hydrophobic interactions and hydrogen bonding were the driving forces for LGO loaded Z-NaCAS NPs formation. In addition, the nanoencapsulated LGO showed a sustained release pattern following Higuchi model as compared to free oil. Regarding the antibacterial activity, both free and nanoencapsulated LGO exhibited considerable activity against the tested bacterial strains, with higher inhibitory effect on Gram-positive bacteria. Mechanistically, the LGO loaded Z-NaCAS NPs demonstrated significantly higher efficacy in bacterial cell membrane disruption, leakage of macromolecules, and interference with AKP and LDH enzymes. In view of that, the present study is putting forth the fabricated Z-NaCAS NPs as a promising nanoplatform for LGO with enhanced physicochemical properties and boosted antibacterial efficacy, eventually expanding its applications in the biomedical and pharmaceutical sectors. Yet, in vivo studies would fortify the current findings in therapeutic contexts.

## Materials and methods

### Materials

Lemongrass leaves (Minnie's Herb^®^, Egypt). Sodium caseinate, zein, and ethanol (95%) were purchased from Sigma Aldrich^®^ (St. Louis, USA). Dimethyl Sulphoxide (DMSO, Gaylord^®^ Chemical Co., LA, USA). Tryptone Soya Agar (TSA, Oxoid^®^). Mueller–Hinton Broth (MHB, Oxoid^®^). Alkaline phosphatase assay kit (MyBioSource^®^, USA). Lactate dehydrogenase assay kit (MyBioSource^®^, USA).

### Extraction of lemongrass essential oil

Dried lemongrass leaves were taxonomically identified as *Cymbopogon citratus* by Prof. Salama Eldarier, Professor of Plant Ecology, Department of Botany, Faculty of Science, Alexandria University, Egypt. Lemongrass leaves (50 g) were washed, chopped into pieces of about 2 cm, and subjected to hydrodistillation for 3 h in a Clevenger-type apparatus. The extracted LGO was then dried over anhydrous sodium sulphate to remove any remaining moisture and stored in dark glass vials at 4 °C for further analysis^69^.

### Phytochemical characterization of lemongrass essential oil

The phytochemical composition of the hydrodistilled LGO was determined by gas chromatography (GC, THERMO Scientific Corp., USA) and mass spectrometry (MS, ISQ Single Quadrupole Mass Spectrometer). A capillary column TR-5 MS column (30 m, 0.32 mm i.d., 0.25 m film thickness) was used, and helium was the transporter gas at a flow rate of 1 mL/min. LGO was diluted to 1 mL with dichloromethane, and 2 μL were then injected in a splitless mode for 1 min, followed by a split flow at a ratio of 1:10. The GC temperature was set at 60 °C for 1 min, increased to 240 °C at a rate of 4 °C per minute, and maintained at 240 °C for 1 min. Temperatures for the injector and detector were both set at 210 °C, and the mass spectra were acquired at 70 eV with a mass range of 40 to 450 m/z. Chemical components of LGO were determined qualitatively using retention indices and spectrum fragmentation patterns matched with MS library databases (NIST and WILEY). Peak area normalization measures were used to quantitatively quantify the constituents of essential oils in terms of the relative percentage of area^70^.

### Preparation of lemongrass oil loaded zein-sodium caseinate nanopatricles (LGO loaded Z-NaCAS NPs)

Lemongrass oil was encapsulated in Z-NaCAS NPs via anti-solvent precipitation method^34^. Zein was dissolved in 80% (v/v) aqueous ethanol solution at a concentration of 25 mg/mL. Lemongrass oil was then added to the Z solution to achieve a LGO:Z mass ratio of 1:5 (w/w). The LGO/Z solution mixture was stirred for 30 min until complete dissolution, after which 2 mL of LGO/Z solution was added dropwise to 10 mL of NaCAS solution under continuous stirring at 600 rpm. The mass ratio of Z/NaCAS biopolymers was set at 2:3 (w/w). The final resultant solution was left overnight under stirring to allow for ethanol evaporation and then freeze-dried at – 50 °C for 48 h.

### Physicochemical characterization of LGO loaded of Z-NaCAS NPs

#### Fourier-transform infrared (FTIR) spectroscopy

The chemical structures and potential interactions of LGO, Z, Na CAS, and developed LGO loaded Z-NaCAS NPs were detected using FTIR spectrophotometer (Perkin Elmer, USA). About 5 mg of each sample was mixed with potassium bromide at a 1:100 ratio and then compressed in the form of discs. The spectra were recorded in the range of 400–4000 cm^−1^ wavenumber^30^.

#### Differential scanning calorimetry (DSC)

The thermal properties of LGO, Z, NaCAS, and fabricated LGO loaded Z-NaCAS NPs were investigated by differential scanning calorimeter (Perkin Elmer, Pyris I, Wellesley, USA). About 5 mg of each respective sample was placed into a sealed aluminium pan. Then, the samples were heated over a range of 20–300 °C at a rate of 10 °C /min under a constant flow of nitrogen gas^71^.

#### Particle size and zeta potential analysis

Particle size distribution, zeta potential (ZP), and polydispersity index (PDI) of blank Z-NaCAS NPs and LGO loaded Z-NaCAS NPs were investigated by dynamic light scattering (DLS) technique using particle size analyzer (Zeta Sizer Pro, Malvern Instruments Ltd., UK) at 25 °C. The dispersion was first diluted with deionized water to avoid multiple scattering effects, and then each sample was analyzed in triplets, and the results were averaged^72^.

#### Encapsulation efficiency and loading capacity

UV–visible spectrophotometry was used to detect the encapsulation efficiency (EE%) and loading capacity (LC%) of LGO in Z-NaCAS NPs. The prepared LGO loaded Z-NaCAS colloidal nanocomplexes were centrifuged at 8000 xg for 30 min to remove the unencapsulated LGO. The obtained supernatant was then diluted with absolute ethanol, and the concentration of unentrapped LGO was measured using UV–visible spectrophotometer (Thermo Scientific^®^, Evolution 300, USA) at ƛ max 230 nm according to a previously established LGO standard calibration curve. The EE% and LC% were calculated based on the following equations. ^26^.

1 $$\text{Encapsulation effeciency (\%) }= \frac{\text{Mass of loaded LGO}}{\text{Mass of initial LGO }}\times 100$$

2 $$\text{Loading capacity (\%) }= \frac{\text{Mass of loaded LGO}}{\text{Total Mass of NPs}+\text{Mass of loaded LGO }}\times 100$$

#### Transmission electron microscopy

The morphological features of LGO loaded Z-NaCAS NPs were visualized using transmission electron microscopy (TEM) (JEM-2000FX, JEOL, Ltd., Tokyo, Japan). At first, the prepared LGO loaded Z-NaCAS NPs were diluted with water, and then one drop of the diluted dispersion was loaded into a copper mesh grid and stained with uranyl acetate solution. After which, the sample-loaded grid was left for air drying, and images were captured at a voltage of 100 kV and a magnification of 50,000^35^.

#### In vitro release kinetics

The release behavior of LGO loaded Z-NaCAS NPs and unencapsulated LGO was investigated using the dialysis bag method. The fabricated Z-NaCAS nanocomplex containing a calculated amount of LGO was placed in a dialysis bag (molecular cut-off of 12–14 kDa MWCO, VISKING dialysis tubing, SERVA, Germany) and suspended in 100 mL of the phosphate buffer saline (pH 7.4), supplemented with 20% ethanol at a temperature of 37 °C with gentle shaking. At a definite time interval, 2 mL of the release medium was taken for analysis and an equivalent amount of the freshly prepared release medium was supplemented to maintain a constant volume. The amount of LGO released from Z-NaCAS NPs was measured using UV–visible spectrometry at λmax 230 nm. The cumulative percentage release of LGO during the 72 h release study was calculated according to the following Equations^73^.

3 $$\text{Cumulative release }(\text{\%}) = \frac{\text{Amount of released LGO at each time interval }}{\text{Total amount of loaded LGO }}\times 100$$

The kinetic release profile of LGO from Z-NaCAS NPs was carried out by fitting the cumulative release data into different kinetic models, including zero-order, first-order, Higuchi, Korsmeyer-Peppas, and Hixson–Crowell models. Then, the data were analyzed according to the linear regression coefficient (R2)^38^. The zero-order model was presented by plotting the cumulative percentage of drug release versus time. To study the first-order model, a graph was plotted between the log % of drug remaining within NPs versus time. The cumulative percentage of drug release versus the square root of time was used to investigate the Higuchi model. To study the Korsmeyer-Peppas model, a graph was created between log cumulative percentage drug release versus log time. To study the Hixson-Crowell model, a graph was plotted showing the cube root of the percentage of drug remaining versus time^50^.

Zero-order model

4 $${\text{C}}_{{\text{t}}} {\text{ = K}}_{{0}} {\text{ t}}$$

where C_t_ is the amount of drug released at time t, K_0_ is the zero-order rate constant and t is the time.

First-order model

5 $${\text{log C}}_{{\text{t}}} {\text{ = log C}}_{{0}} {\text{ - K}}_{{1}} {\text{ t/2}}{.303}$$

where C_t_ is the percent of drug remaining at time t, C_0_ is the initial concentration of the drug, K_1_ is the first-order rate equation expressed in time^−1^.

Higuchi model

6 $$\text{Q} = \text{K}_\text{H} \times \text{t}^{1/2}$$

where Q is the cumulative amount of drug released in time t per unit area and K_H_ is the Higuchi dissolution constant.

Korsmeyer–Peppas model

7 $$M_{t} /M_{\infty } = K_{KP} \times t^{n}$$

where M_t_/M_∞_ is the amount of drug released at time t, K_KP_ is the rate constant and n is the release exponent.

Hixson–Crowell model

8 $${\text{W}}_{{0}}^{{1/3}} {\text{ - W}}_{{\text{t}}}^{{1/3}} {\text{ = K}}_{{{\text{HC}}}}^{{\text{t}}}$$

where W_0_ is the initial amount of drug in the nanoparticles, W_t_ is the remaining amount of drug in the nanoparticles at time t and K_HC_ is the Hixson–Crowell constant.

### Antibacterial activity

#### Bacterial strains

The antibacterial activity of free LGO and LGO loaded Z-NaCas NPs was evaluated using four reference bacterial strains, including *Staphylococcus epidermidis* (ATCC 12,229), *Enterococcus faecalis* (ATCC 29,122), *Escherichia coli* (ATCC 25,922), and *Klebsiella pneumoniae* (ATCC 700,603). The tested bacteria were maintained on TSA and stored at 4 ^◦^C.

#### Minimum inhibitory concentration (MIC)

The broth microdilution method was used to determine the minimum inhibitory concentration of blank Z-NaCAS NPs, free LGO and LGO loaded Z-NaCAS NPs^74^. Overnight MHB cultures of the corresponding bacterial strain were standardized to 0.5 McFarland turbidity standard (10^6^ CFU/ mL). Stock solutions of free LGO in DMSO, and LGO loaded Z-NaCAS NPs were prepared at concentrations of 3 mg/mL. Two-fold serial dilutions (1.5–0.012 mg/mL) of the stock solutions were performed in a 96-well microtiter plate containing MHB. Inoculated MHB was used as a growth control, and inoculated MHB with either blank Z-NaCAS NPs or DMSO was used as a vehicle control, while uninoculated MHB was used as a sterility control. The MIC was determined as the lowest concentration of the respective tested agent that suppressed observable bacterial growth after 18 h incubation at 35 °C.

### Antibacterial mechanisms

#### Scanning electron microscopy (SEM)

The morphological changes induced by the formulated LGO loaded Z-NaCAS micelles were examined using SEM^58^. Cultures of *S. epidermidis*, *E. faecalis*, *E. coli*, and *K. pneumoniae* standardized to 0.5 McFarland were treated with 1 MIC of the prepared nanoformulation for 4 h. Each respective culture was subjected to centrifugation at 4000 xg for 10 min, followed by washing three times with PBS (pH 7.4). Both control and treated cultures were fixed with 2.5% glutaraldehyde for 4 h at 4 °C, dehydrated in graded ethanol (50–100%), and finally sputtered with gold prior to investigation by SEM (JSEM-IT 200, JEOL, Ltd., Tokyo, Japan).

#### Cell membrane integrity assay

The effect of LGO loaded Z-NaCAS NPs and free LGO on the bacterial membrane was investigated by assessing the leakage of proteins and nucleic acids^75^. Each respective overnight bacterial culture was adjusted to 0.5 McFarland turbidity standard, centrifuged at 4000 xg for 10 min, and the obtained pellets were washed three times and resuspended in sterile PBS. *S. epidermidis, E. faecalis, E. coli,* and *K. pneumoniae* cultures were treated with 1 MIC of free or LGO loaded Z-NaCAS NPs and incubated at 37 °C for 2 h with shaking. Control and treated bacterial cultures were subjected to centrifugation at 8000 xg for 5 min, and the absorbance of cellular components in the supernatant was assessed at λ max of 260 nm and 280 nm as an indicator for genetic materials and protein release using UV–visible spectrophotometry.

#### Lactate dehydrogenase activity assay

Lactate dehydrogenase (LDH), an intracytoplasmic enzyme, was assessed as an index for the antibacterial inhibitory effect of the fabricated nanoformulation^76^. The activity of LDH was assayed using an LDH assay kit according to the manufacturer’s procedure. The supernatant of the respective bacterial strain treated with 1 MIC of either free or LGO loaded Z-NaCAS NPs was tested at λmax of 450 nm using UV–visible spectrometry following centrifugation at 5000 xg for 10 min.

#### Alkaline phosphatase activity assay

Alkaline phosphatase (AKP) is a periplasmic enzyme, and its leakage is an index for the integrity of the bacterial cell wall. The release of AKP in the culture supernatant of tested bacterial strains was tested using an AKP assay kit^77^. The overnight culture of the respective bacterial strain was standardized to 0.5 McFarland turbidity standard and then incubated with 1 MIC of free or LGO loaded Z-NaCAS NPs for 2 h at 37 °C. Following incubation, the tested bacterial cultures were centrifuged at 5000 xg for 10 min, and the AKP activity was assessed using the aforementioned AKP kit as per the manufacturer’s procedure.

### Statistical analysis

All measurements were done in triplicate and presented as mean ± standard deviation (SD). The results were statistically analyzed by one-way analysis of variance (ANOVA), followed by Tukey’s post hoc multiple comparison test by using GraphPad Prism version 8.0.2 software. The values of *P < 0.05, **P < 0.01, and ***P < 0.001 were considered statistically significant.

### Supplementary Information

Supplementary Information.

## Data Availability

All data in this study are publicly available and the raw analysis data can be obtained by contacting the corresponding author upon request.
