# Supplementary material for: Enhancement of lemongrass essential oil physicochemical properties and antibacterial activity by encapsulation in zein-caseinate nanocomposite
Source: Sci Rep. 2024 Jul 27;14:17278. doi: 10.1038/s41598-024-67273-6 (PMC11283490; doi:10.1038/s41598-024-67273-6)
Supplement: Supplementary file 1 — Supplementary Information. [file 41598_2024_67273_MOESM1_ESM.docx]

**Enhancement of lemongrass essential oil physicochemical properties and antibacterial activity by encapsulation in zein-caseinate nanocomposite**

**Sara A. Alsakhawy ^a*^, Hoda H. Baghdadi ^a^, Moustafa A. El-Shenawy ^b^, Lobna S. El-Hosseiny ^a^**

^a^ *Department of Environmental Studies, Institute of Graduate Studies and Research, Alexandria University, Alexandria 21526, Egypt*

^b^ *Department of Food Microbiology, National Research Center, Dokki, Cairo 12311, Egypt*

Corresponding author:

Dr. Sara A. Alsakhawy

E-mail address: [igsr.sarahatef@alexu.edu.eg](mailto:igsr.sarahatef@alexu.edu.eg)

Orcid ID: 0000-0002-4856-9824

**Supplementary Table S1:** Release kinetics of LGO from Z-NaCAS NPs in phosphate buffer saline media (pH 7.4)

| **Nanoparticles** | **Zero-order** | **First-order** | **Korsmeyer-Peppas** | | **Higuchi** | **Hixson-Crowell** |  |
| --- | --- | --- | --- | --- | --- | --- | --- |
|  | **R2** | **R2** | **R2** | **n** | **R2** | **R2** |  |
| **LGO-Z-NaCAS** | 0.90 | 0.94 | 0.95 | 0.45 | 0.98 | 0.93 |  |

Where R2 is the correlation coefficient and n is the diffusion exponent.

| **a) Zero-order kinetic release model** | **b) First-order kinetic release model** |
| --- | --- |
| 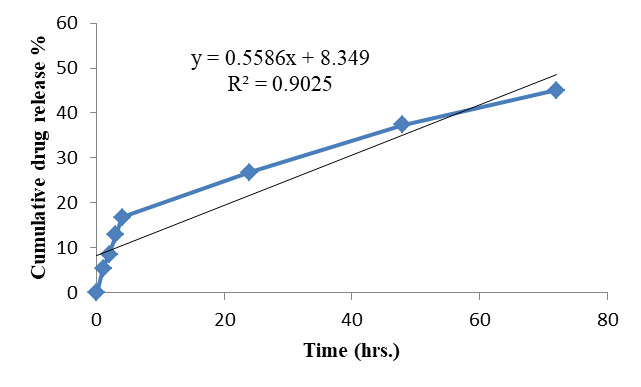 | 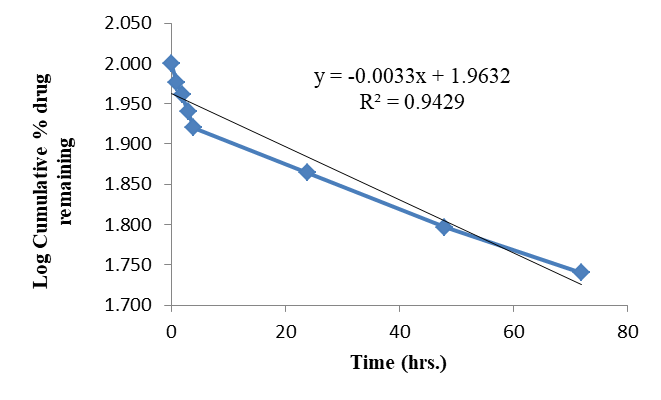 |
| **c) Higuchi kinetic release model** | **d) Korsmeyer-Peppas kinetic release model** |
| 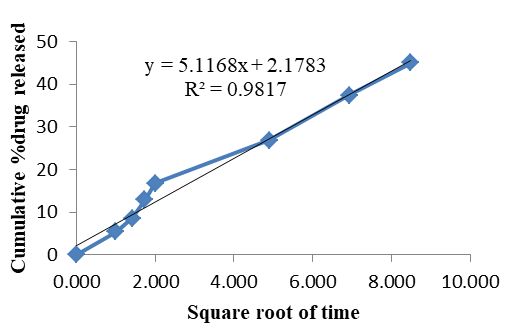 | 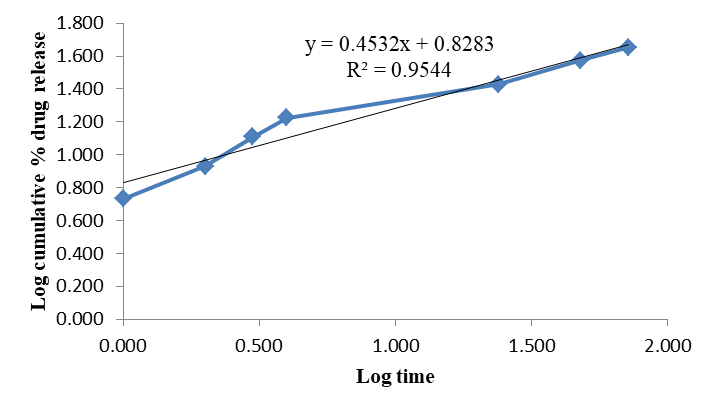 |
| **e) Hixson-Crowell kinetic release model** | |
| 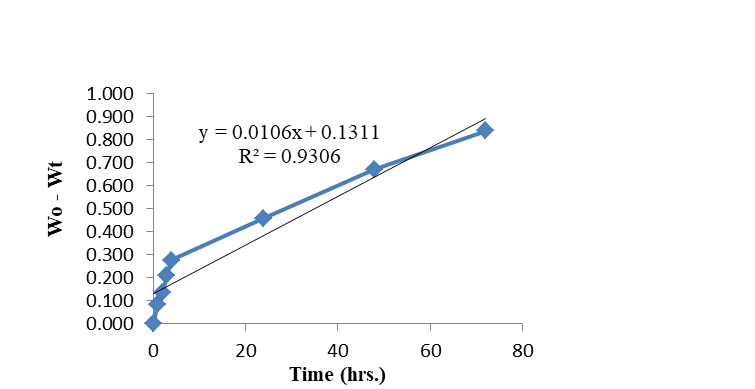 | |

**Supplementary Figure S1:** Drug release kinetic plots: a) Zero-order, b) First-order, c) Higuchi, d) Korsmeyer-Peppas and e) Hixson-Crowell
